# Droplet distribution and mitigation of occupational exposure risk in eucalyptus sprout eradication using a remotely piloted aircraft

**DOI:** 10.3389/fpls.2024.1504608

**Published:** 2025-01-17

**Authors:** Luis Felipe Oliveira Ribeiro, Edney Leandro da Vitória, Halisson Pereira Bastos, Jacimar Vieira Zanelato, José de Assis Martins Júnior, Alexandre de Vicente Ferraz, Thales Gomes dos Santos, Francisco de Assis Ferreira, João Victor Oliveira Ribeiro, Samuel de Assis Silva, Pengchao Chen

**Affiliations:** ^1^ Department of Agricultural and Biological Sciences (DCAB), Federal University of Espirito Santo (UFES), São Mateus, ES, Brazil; ^2^ Postgraduate Program in Tropical Agriculture (PPGAT), Federal University of Espirito Santo, São Mateus, ES, Brazil; ^3^ Emflora Forestry Services and Enterprises, Remote Aeroagricultural Department, São Mateus, ES, Brazil; ^4^ Institute of Forest Research and Studies (IPEF), Piracicaba, SP, Brazil; ^5^ Department of Rural Engineering, Federal University of Espírito Santo, Alegre, ES, Brazil; ^6^ National Center for International Collaboration Research on Precision Agricultural Aviation Pesticides Spraying Technology, College of Electronic Engineering and Artificial Intelligence, South China Agricultural University, Guangzhou, China

**Keywords:** eucalyptus ssp., forestry, droplet deposition, application technology, risk of exposure, spray drift, unmanned aerial spraying system, unmanned aerial vehicle

## Abstract

The use of remotely piloted aircrafts (RPAs) for foliar application of pesticides and fertilizers has increased worldwide in several agricultural crops. However, there is little information on the efficiency and factors connected to application and spraying quality of RPAs in forestry, mainly for eradication of eucalyptus sprouts. The objective of this work was to evaluate droplet distribution and deposition on eucalyptus sprouts and the risk of exposure for applicators using an RPA (DJI AGRAS T40) at different theoretical application ranges (7.0, 9.0, and 11.0 m) and droplet sizes (150, 300, and 450µm) compared to a manual electric backpack sprayer (MEBS). The spray solution was composed of water, brilliant blue dye, and adjuvant. Water-sensitive paper cards and flexible polyvinyl chloride cards were positioned on different eucalyptus sprout canopy layers (ESCL) (upper, middle, and lower) to evaluate droplet distribution and deposition. Disposable coveralls, gloves, and respirators were used to evaluate the risk of occupational exposure. The results showed that the application ranges of 7.0 and 9.0 m with droplet sizes of 150µm and 300µm resulted in better droplet distribution throughout the ESCLs. However, the 450µm droplet size resulted in concentration of droplets in the upper ESCL. Using an MEBS resulted in greater heterogeneity in droplet distribution and approximately a 160-fold higher accumulation of droplets on different applicator’s body parts compared to the RPA. The results confirmed the efficiency and operational safety of using RPAs for the application of agricultural pesticides and foliar fertilizers in eucalyptus plantations, as well as providing valuable contributions for future research on these practices in eucalyptus cultivation.

## 1 Introduction

Eucalyptus (*Eucalyptus* spp.) is the main planted forest species worldwide due to its economic importance in the market of forestry products, ecological and environmental safety, and mitigation of climate change (Palma et al., 2021; Tang et al., 2023). The main producing countries include Brazil, China, India, and Australia. Eucalyptus plantations in Brazil have high production potential, estimated in 33.7 m^3^ ha^-1^ year^-1^ (with bark) and are grown in approximately 7.83 million hectares (IBÁ, 2024).

The growth, development, yield, and quality of products from fast-growth forests, as eucalyptus, are affected by the interactions between environmental, genetics, and management factors over the production cycle. The reforesting process in these plantations requires the planting of new seedlings after the mature forest harvest. Therefore, the eradication of undesired sprouts that emerge from epicormic and lignotubers in the base of trees is essential to ensure the growth of new seedlings and minimize competition for resources (water, light, nutrients and space) (Burrows, 2013; Clarke et al., 2013). These sprouts persist in stumps after the timber harvest and removing them for pre-establishment of new plantations is crucial (Montes et al., 2022). Chemical control through application of herbicides with systemic and contact modes of action is one of the main approaches for removing these sprouts (Ferreira et al., 2010).

Conventional methods of pesticide application in forestry usually involve the use of ground sprayers, as backpack and tractor-mounted sprayers (boom, self-propelled, pneumatic, and hydropneumatic). Mewes et al. (2013) highlight that ground applications in forests favor intensive monitoring and localized control of biological target; moreover, they showed that aerial applications by airplanes results in droplets being predominantly directed towards the upper canopy layer, causing difficulties to reach targets in lower canopy layers. Therefore, ensuring the uniform distribution of the active ingredient over, covering the entire target canopy of plants or, at least, the predominant areas of biological targets is essential to obtain a higher efficiency in plant protection in forests (Pachuta et al., 2023).

The use of remotely piloted aircraft (RPA) is increasingly expanding in the agriculture and forestry sectors. RPAs can be classified into two large groups: RPAs for aero surveys, focused on photogrammetry using optical and thermal sensors; and RPAs for agricultural purposes, as pesticide and foliar fertilizer applications and dispersion of solids, as mineral fertilizers and seeds. Studies using RPAs on eucalyptus plantations are currently concentrated on forest monitoring (Marques et al., 2024), estimating of trunk diameters (Prabhu et al., 2024) and plant aerial biomass (Liu et al., 2023), detection of trees and qualitative inventory (Almeida et al., 2021), detection of foliar diseases (Liao et al., 2022), detection and measurement of cutter-ant nests (Santos et al., 2022), detection of planting flaws (Zhao et al., 2021). However, there is a significant lack of information regarding the use of RPAs for pesticide application in forestry areas.

The technology of application of pesticides and foliar fertilizers using RPAs stands out by its advantages compared to conventional sprayers, including higher operational efficiency, low application rates, water economy, lower risk of contamination of applicator, and fit for mountainous terrain and muddy fields; in addition, it does not require takeoff or landing tracks and is not limited by topography, space for turning, and canopy of target plants (Wang et al., 2019; Biglia et al., 2022; Mahmud et al., 2023; Arakawa and Kamio, 2023). However, some current limitations include the demand for specialized labor, operational time due to battery life, and increased risk of drift under inadequate operational parameters (flight height and operational speed, application rate, spray nozzles, flight path, and application range) and weather conditions during applications (Richardson et al., 2020; Guo et al., 2020; Gugan and Haque, 2023; Carneiro et al., 2024).

Studies on the control of pests, diseases, and weeds using RPAs are concentrated in annual crops, such as rice (Chen P. et al., 2020), cotton (Wang G. et al., 2022), maize (Shan et al., 2022), soybean (Lopes et al., 2023), wheat (Pranaswi et al., 2024), and perennial plants, such as apple (Wang C. et al., 2022), citrus (Nascimento and Vitória, 2022), vine (Biglia et al., 2022), peach (Li et al., 2022), Brazil nut (Arakawa and Kamio, 2023), coffee (Vitória et al., 2023), almond (Li et al., 2021), coconut (Lan et al., 2024), and olive (Martinez-Guanter et al., 2020). However, specific morphological characteristics of eucalyptus, as erect growth habit, rounded canopy architecture, and ascending branches from the trunk, poses unique challenges for the planning of pesticide applications, different from those of other agricultural crops, emphasizing the need of adapted approaches to eucalyptus plantations.

One of the main problems to be minimized during the applications with RPAs is the movement of droplets directed to areas outside the target due to air flow, called drift (Wang et al., 2018). The exposition of applicators to pesticides can occur, regardless of the application method used (ground or aerial), mainly by inhalation and dermal contact during the mixture, loading, cleaning of equipment, entry in treated areas, and spray drift (Pinto et al., 2020; Yan et al., 2021). Although RPA operators maintain a distance from the equipment during pesticide applications, the combination of fine droplets, the high distance between the spray nozzle and the target, and unfavorable weather conditions can result in direct and indirect exposure of individuals near the treated areas; additionally, the solutions applied by RPAs are extremely concentrated (Wang et al., 2023; Dubuis et al., 2023). Previous studies have highlighted the need for real-time monitoring of occupational exposure risk for applicators and spray quality on target plants during RPA applications, as recently shown in shrub fruit orchards by Dubuis et al. (2023); Wang et al. (2024), and Lan et al. (2024).

Therefore, the hypotheses raised in this study were: (a) the interaction between theoretical application range and droplet size affects the quality of applications to eucalyptus plantations; (b) the risk of occupational exposure for applicators is increased by decreasing the size of the sprayed droplets; (c) the distribution of droplets sprayed using RPAs has the potential for quality in eucalyptus sprout eradication with herbicides; and (d) the spray uniformity achieved using RPAs is greater than that obtained with a manual electric backpack sprayer.

In this sense, the objective of this work was to evaluate the distribution and droplet deposition on eucalyptus sprouts and the risk of exposure for applicators using an RPA (DJI AGRAS T40) with centrifugal nozzles, using different operational parameters (application range and droplet size) compared to a manual electric backpack sprayer.

## 2 Material and methods

### 2.1 Characterization of the study area

The experiment was conducted in a commercial eucalyptus area in São Mateus, state of Espírito Santo, Brazil (18°34’02’’S; 40°05’10”W). The soil of the area was classified as Typic Hapludult of loamy sandy texture. The region’s climate was classified as Aw, hot and humid, with a dry season in the autumn-winter and a rainy season in the spring-summer, according to the Köppen classification (Alvares et al., 2013).

**Figure 1:**
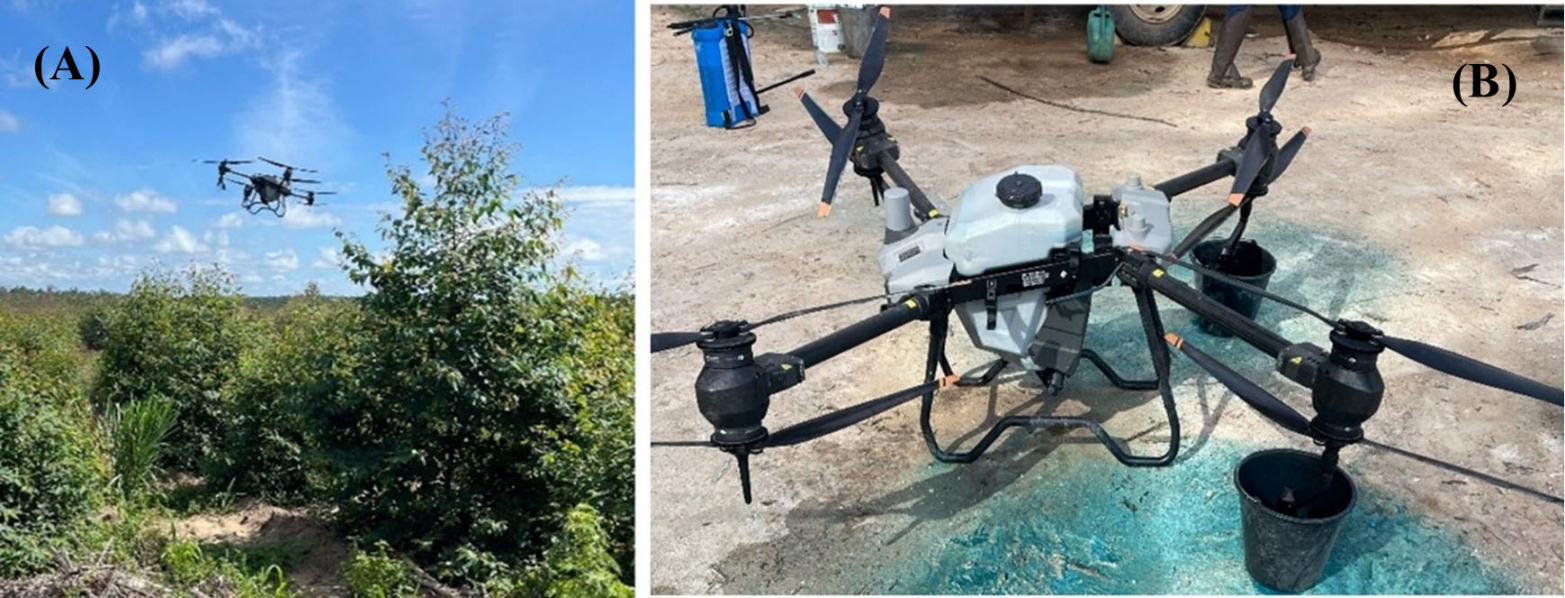
Remotely piloted aircraft (RPA) (DJI Agras T40; SZ DJI Technology, Nanshan, China) used in the experiment: **(A)** RPA flying over the experimental area); **(B)** Regulation and calibration prior to the operations using a brilliant blue dye tracer.

The experimental area was composed by eucalyptus sprouts (hybrid *Eucalyptus grandis* × *Eucalyptus urophylla*, commercial clone CCARAOP), with mean height of 2.80 m (measured with a tape measure from the stem base to the apex) and spacing of 3.60 × 2.50 m, totaling 1,111 plants per hectare. **Figure 1** shows the location of experimental area and the distribution of eucalyptus plants.

### 2.2 Sprayer devices

The remotely piloted aircraft (RPA) (DJI Agras T40 model; SZ DJI Technology, Nanshan, China) used in the experiment (**Figure 1**) was set and calibrated prior to the applications. This RPA features a coaxial dual-rotor structure, with eight rotors mounted above and below the four articulating arms. The spraying system is formed by a magnetically-driven centrifugal pump, a flow controller, and two atomized nozzles with anti-dripping centrifugal valves that have a theoretical droplet size adjustment from 50 to 500 µm.

The following operational parameters were maintained constant in the RPA for all treatments: application rate of 12.0 L ha^-1^, flight speed of 5.0 m s^-1^, operational flight height of 4.0 m above the eucalyptus sprout canopy; and application route parallel to the planting rows, considering the wind perpendicular to the aircraft direction.

The manual electric backpack sprayer (MEBS) used (Yamaho^®^; Mogi das Cruzes, Brazil) had a solution tank capacity of 20 L and a Yamaho SR-1 flat fan nozzle with air induction. The application rate used was approximately 100 L ha^-1^. The spraying quality was standardized with applications at an operational speed of approximately 1.0 m s^-1^. The working pressure was 200 kPa, resulting in a flow rate of 0.65 L min^-1^ and an application range of 1.05 m.

### 2.3 Experimental design

The experiment was divided into two phases. The first phase consisted of obtaining data on application quality and droplet deposition on eucalyptus sprouts, whereas the second phase consisted of determining the risk of occupational exposure for applicators during the experimental applications.

The experiment was conducted in a randomized block design with four replications, with treatments implemented in a 3×3 + 1 factorial arrangement, consisting of three droplet sizes (150, 300, and 450µm); three application ranges (7, 9, and 11 m), defined in the RPA control settings; and a control treatment (using the MEBS). After the repetitions of each experimental treatment, the adjustments of the operational parameters (application range and droplet size) were manually made in the RPA control system. **Table 1** shows the experimental treatments.

**Table 1:** Experimental treatments.

Treatments	Application range (m)	Droplet size (µm)	Droplet classification
T1	7.0	150	Fine
T2	9.0	150	
T3	11.0	150	
T4	7.0	300	Mean
T5	9.0	300	
T6	11.0	300	
T7	7.0	450	Very coarse
T8	9.0	450	
T9	11.0	450	
T10	Manual electric backpack sprayer		Extremely coarse

Source: ASABE S572.1 (ASABE, 2020) and ISO 25358:2018 (ISO, 2018).

The experimental area was 6,400 m² (80 × 80 m). All treatments were carried out in total area and in the same block (application day). A 15.0 m border area was disregarded at the edges to allow some distance for the RPA sprayer to be activated.

**Figure 2:**
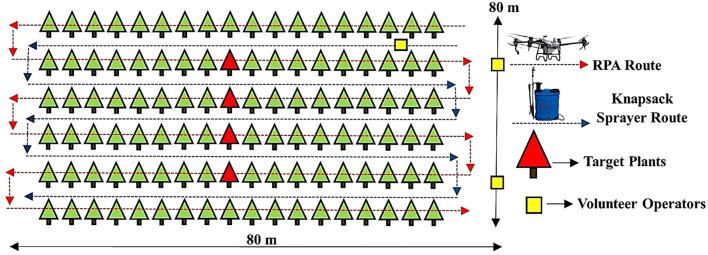
Experimental design of the area with position of artificial targets and volunteers.

The experimental unit (central area) was 2,400 m² (80 × 35 m), where the eucalyptus sprouts had a greater uniformity in relation to the canopy shape. The data collection points in each treatment were four target plants, spaced approximately 3.60 m apart, that together composed the experimental unit. For the second phase, two volunteer applicators stayed at 15.0 m from the end of the RPA application route in the experimental area, spaced 60.0 m apart. The treatment using the backpack sprayer was performed by a volunteer applicator passing between the planting rows to apply the solution on eucalyptus sprouts. **Figure 2** shows the experimental area design.

The solution sprayed in all treatments in both experimental phases was composed of water, brilliant blue dye at the rate of 4.0 g L^-1^, and non-silicone adjuvant based on balanced polymers specific for aerial applications with low solution volume (0.2% v v^-1^) (Helper Air^®^, ICL, São Paulo, SP, Brazil).

### 2.4 Determination of application quality

**Figure 3:**
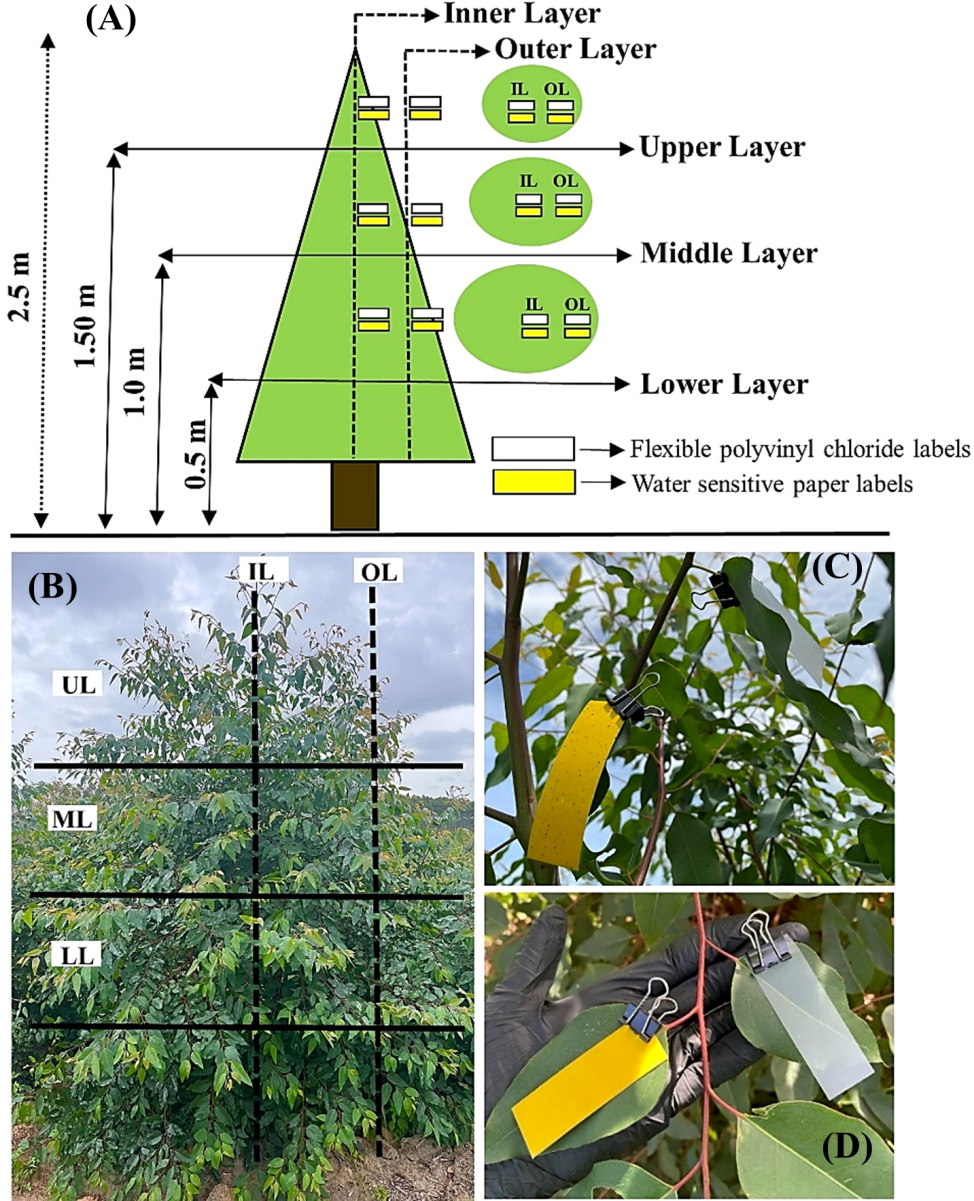
(**A**, **B**) Positioning of water-sensitive paper cards and flexible polyvinyl chloride cards on the inner (IL) and outer (OL) sections of the upper (UL), middle (ML), and lower (LL) layers of the eucalyptus sprout canopy; (**C**, **D**) positioning according to the angle and alternate phyllotaxy of eucalyptus leaves.

Application quality was characterized using water-sensitive paper cards (76 × 26 mm), and droplet deposition was evaluated using rectangular flexible polyvinyl chloride cards (76 × 26 mm). Water-sensitive paper and flexible polyvinyl chloride cards were attached to each target plant with the aid of metal clips, positioning them at the same height in relation to the canopy, following the angle and alternate phyllotaxy of leaves in the inner and outer sections of the upper, middle, and lower layers of eucalyptus sprout canopies (**Figure 3**). The positioning of both card types was determined focusing on the overlap of droplets in each experimental treatment.

Both card types were collected five minutes after application of each treatment, using powder-free nitrile gloves, to allow the solution to evaporate, maintaining only the dye. They were then placed in kraft paper bags (water-sensitive paper cards) or labeled plastic bags (flexible polyvinyl chloride cards), which were labeled. These samples were then placed in expanded polystyrene boxes and taken to the Laboratory of Mechanization and Agricultural Defensives (LMDA) of the Northern Espírito Santo University Center of the Federal University of Espírito Santo, in São Mateus, ES, Brazil for analyses.

A wireless system (DropScope^®^; SprayX, São Carlos, Brazil) was used to scan the water-sensitive paper cards for data acquisition and analysis. This system consists of an application software and a wireless digital microscope equipped with a digital image sensor with a resolution of over 2,500 dpi, allowing for the detection of partially overlapping droplets with a diameter of approximately 25µm. Recent research studies have confirmed the reliability of data obtained using this system for assessing spectra of droplets sprayed on shrubby crops by RPAs (Vitória et al., 2023; Lopes et al., 2023; Santos et al., 2024; Ribeiro and Vitória, 2024).

The following parameters were obtained through water-sensitive paper cards: droplet coverage (%); droplet density (droplets cm^-2^); volume distribution by droplet size class (Dv_0.5_ or VMD, Dv_0.1_, and Dv_0.9_, i.e., diameters of droplets composing 50%, 10%, and 90% of the applied volume, respectively); relative amplitude, a coefficient that determines droplet uniformity (dimensionless); and potential drift risk (%), which is the percentage of the applied volume composed of droplets smaller than 100µm.

### 2.5 Droplet deposition

Droplet deposition was estimated by removing the dye in the laboratory, washing the flexible polyvinyl chloride cards with 50 mL of deionized water per sample. The samples were manually shaken for approximately 60 seconds; the resulting solutions were subjected to absorbance readings at 630 ηm on a digital UV-VIS spectrophotometer with a holographic flashing monochromator (IL-226-NM-BI; Kasuaki^®^, China).

The development of the calibration curve was based on absorbance of seven solutions with standard concentrations (0, 2.5, 5, 10, 20, 25, and 100 ppm). The analysis of the linear relationship between the concentration and the absorbance of standard solutions resulted in a coefficient of correlation of R^2^ = 0.999. The equation that described this correlation was *y = 0.0008x + 0.0015*. Droplet deposition per unit area (µL cm^-2^) was calculated considering the spectrophotometer readings, the calibration curve data, and area of flexible polyvinyl chloride cards, as described by Vitória et al. (2022); Crause et al. (2023), and Ribeiro et al. (2023a).

### 2.6 Determination of occupational exposure of applicators

The solution described in section 2.4. was applied as a substitute of pesticides to measure and compare the occupational exposure of applicators to droplets applied by an RPA and an MEBS, using operational conditions identical to those described in section 2.2. The experimental treatments were similar to those described in section 2.3.

The test with the RPA was conducted with two volunteer applicators (mean height of 1.70 m and mean weight of 70 kg), who stayed 60.0 m distant from each other, at 15.0 m from the RPA flight path in the experimental area, as shown in the experimental setup in section 2.3 (Experimental design). The test with the backpack sprayer was carried out with the selection of an applicator with technical experience in spraying, as the applications of herbicides in forest areas; the applicator with a lance held in the right hand carried out up and down movements of 0.50 m over the canopy of plants, moving forward continuously through the area to be sprayed.

**Figure 4:**
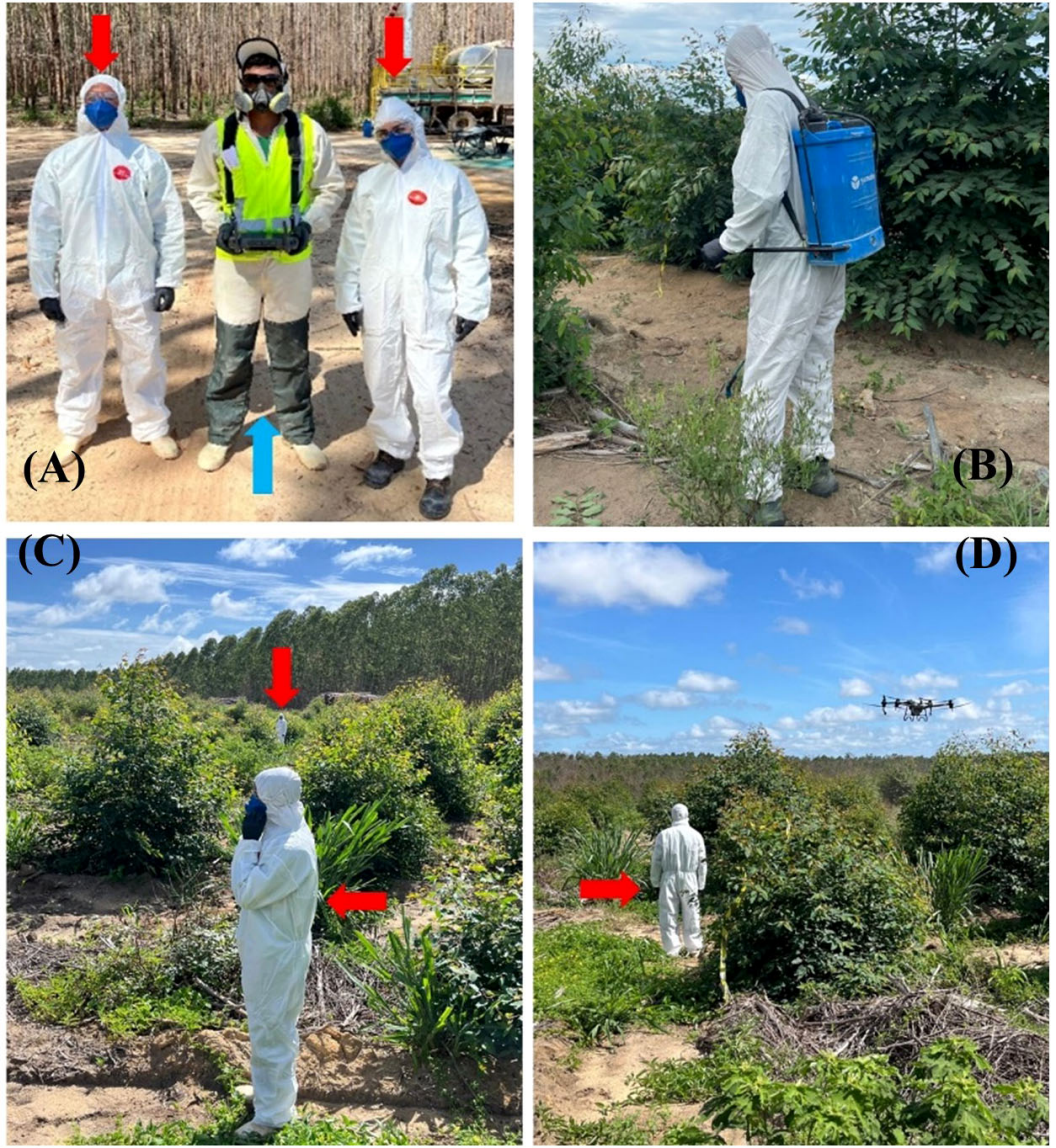
**(A)** volunteer applicators during simulated exposure (red arrows) to treatments using remotely piloted aircraft (RPA); RPA operator (blue arrow) wearing the required personal protective equipment, as described in Ordinance n°. 298/2021 of the Brazilian Ministry of Agriculture and Livestock (MAPA); **(B)** applicator using a manual electric backpack sprayer, equipped with disposable coveralls, nitrile gloves, respirators, and transparent safety glasses; (**C**, **D**) volunteer applicator (red arrow) exposed and positioned during RPA treatment applications.

The potential for dermal occupational exposure of applicators was determined using whole-body dosimetry, as described in previous studies (Tsakirakis et al., 2014; Cao et al., 2015; Yan et al., 2021; Lake et al., 2021). Prior to each experimental treatment, the volunteer applicators (RPA and MEBS) were dressed on their everyday clothing, disposable coveralls with micropore laminated tissue hood (Steelflex^®^, São Paulo, Brazil), a pair of disposable powder-free nitrile gloves with texturized fingertips, a disposable valveless respirator with synthetic fiber filters, and safety transparent glasses produced according to the ANSI Z87.1-2003 standard (**Figure 4**). The two volunteer applicators corresponded to two replications per treatment for each experimental RPA treatment, as they were using new items (disposable coveralls, gloves, and respirators) in each experimental treatment to determine their individual dermal exposure.

**Figure 5:**
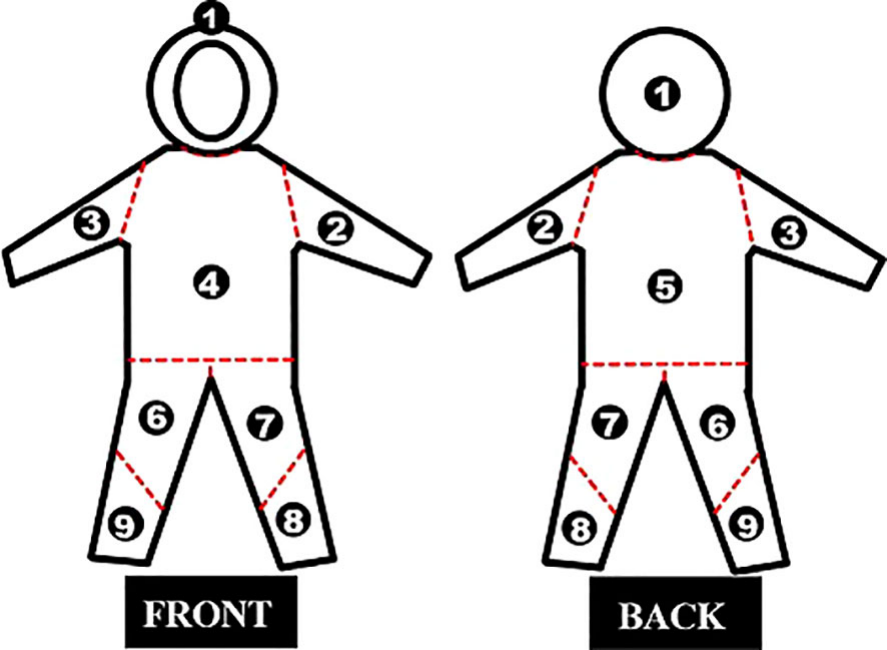
Sectioning of disposable coveralls for analysis of whole-body dosimetry: (1) head, (2) left arm, (3) right arm, (4) chest, (5) back, (6) right thigh, (7) left thigh, (8) left leg, and (9) right leg.

Approximately 15 minutes after the application of each treatment, to allow for the evaporation and drying of spray drift droplets, the disposable coveralls, gloves, and respirators were removed carefully by two assistants using nitrile gloves to avoid cross contamination. The disposable coveralls were cut with scissors, sectioning them nine parts corresponding to different parts of the applicator body: head, left arm, right arm, chest, back, right thigh, left thigh, left leg, and right leg (**Figure 5**). Samples of sectioned parts of disposable coveralls, gloves, and respirators, were placed in labeled plastic bags, individually sealed, taken to the laboratory, and refrigerated until residual dye extraction.

In the laboratory, sections of disposable coveralls, gloves, and respirators were subjected to the same procedure described in section 2.4. (Determination of application quality) for determination of dye deposition, using spectrophotometry. However, the area of each part of disposable coveralls was measured with a ruler, resulting in the following measurements: 1,080 cm² (legs), 960 cm² (thigh), 3,998 cm² (chest), 1,518 cm² (arms), 705 cm² (head), 330 cm² (respirator), and 680 cm² (hands). Each part was carefully withdrawn from the plastic bags using laboratory tweezers and individually placed in a 2,0 L glass backer. The coverall parts 1-9 were washed using 1,0 L of deionized water, and each glove and respirator was washed using 400 mL of deionized water. The bakers were shaken every minute for ten minutes with aid glass rod to ensure complete extraction of deposited droplets; the solutions were then subjected to spectrophotometric readings.

### 2.7 Monitoring of weather conditions during applications

The applications were carried out in the mornings. Weather data were recorded by a meteorological station (5500AG; Kestrel^®^, Boothwyn, USA). Air temperature during the experiment varied between 25.7 and 29.1°C, with a mean relative air humidity exceeding 55%, and mean wind speed between 1.3 and 5.8 km h^-1^. The methodology described in the ISO Standard 22866 of the International Organization for Standardization (ISO, 2005) establishes that air temperature during applications should be between 5 and 35°C, with a maximum of 10% of wind speed measurements below 1.0 m s^−1^, and a wind direction limit of 90 ± 30° in relation to the application line.

### 2.8 Statistical analysis

The Shapiro-Wilk test was applied to evaluate the homogeneity and normality of the data residuals. The results of application quality and droplet deposition were then subjected to the Tukey’s test for pairwise comparisons, and the Dunnett’s test was used for comparisons with the control treatment (MEBS) when needed. All statistical analyses were conducted in the Rbio statistical software at a 5% significance level (Bhering, 2017).

## 3 Results

The results found for all variables evaluated in the inner and outer sections of the eucalyptus sprout canopy layers (ESCL) (upper, middle, and lower ESCL) were not significantly different. The interaction effect between the factors was not significant for any ESCL (p ≥ 0.05). Thus, the factors evaluated for the remotely piloted aircraft (RPA) (application range and droplet size) were analyzed separately.

### 3.1 Drop coverage, drop density, drop deposition, and the potential risk of drift

Droplet size and application range significantly affected droplet density and potential drift risk when using RPA for all ESCLs. Droplet deposition on all canopy layers was not significantly affected by the factors, confirming the null hypothesis of no difference between treatments (**Table 2**).

**Table 2:** Analysis of variance for droplet coverage (%), droplet density (droplets cm^-2^), droplet deposition (µL cm^-2^), and potential drift risk (%) on eucalyptus sprout canopy layers (ESCL) (upper, middle, and lower) when using a remotely piloted aircraft.

Factor	Droplet coverage (%)			Droplet density (droplets cm^-2^)		
	Upper	Middle	Lower	Upper	Middle	Lower
Range (R)	0.30^ns^	0.97^ns^	0.10^ns^	<0.01^**^	<0.05^*^	<0.05^*^
Droplet (D)	0.14^ns^	0.17^ns^	<0.05^*^	<0.001^***^	<0.05^*^	<0.05^*^
R× D	0.73^ns^	0.54^ns^	0.08^ns^	0.06^ns^	0.18^ns^	0.08^ns^
CV (%)	72.14	67.32	61.06	55.57	84.45	84.56
Factor	Droplet deposition (µL cm^-2^)			Potential drift risk (%)		
	Upper	Middle	Lower	Upper	Middle	Lower
Range (R)	0.41^ns^	0.40^ns^	0.97^ns^	<0.05^*^	<0.01^**^	<0.01^**^
Droplet (D)	0.43^ns^	0.47^ns^	0.26^ns^	<0.05^*^	<0.001^***^	<0.05^*^
R × D	0.69^ns^	0.49^ns^	0.62^ns^	0.83^ns^	0.56^ns^	0.78^ns^
CV (%)	72.86	31.84	55.58	58.72	68.90	72.12

Range = application range (m); Droplet = droplet size (µm); R × D = interaction between application range and droplet size. * = significant at p < 0.05; ** = significant at p < 0.01; *** = significant at p < 0.001; ns = not significant; CV (%) = coefficient of variation.

The 9.0 m application range resulted in greater mean droplet coverage for all ESCLs, presenting means 15.09% and 30.18% higher than those found for application ranges of 7.0 and 11.0 m, respectively (**Table 3**). However, these differences were not statistically significant. Droplet size was significantly affected by the application range only for the lower ESCL, in which 300µm droplet size had the highest mean coverage. Despite the variations in mean droplet coverage among the ESCLs, according to the different droplet sizes, the overall means for 150, 300, and 450µm were similar (**Table 3**).

**Table 3:** Mean droplet coverage (%) and droplet density (droplets cm^-2^) on the upper, middle, and lower layers of the eucalyptus sprout canopy based on different application treatments using a remotely piloted aircraft (RPA) and a manual electric backpack sprayer (MEBS).

	Droplet coverage (%)				Droplet density (droplets cm^-2^)			
RPA application range (m)								
	Upper	Middle	Lower	Mean	Upper	Middle	Lower	Mean
7.0 m	0.63 a	0.40 a	0.33 a	0.45	20.78 a	9.27 a	6.76 ab	12.27
9.0 m	0.75 a	0.43 a	0.42 a	0.53	16.93 a	10.37 a	10.84 a	12.71
11.0 m	0.46 a	0.42 a	0.22 a	0.37	9.0 b	5.74 b	4.05 b	6.26
RPA droplet size (µm)								
150µm	0.76 a	0.41 a	0.24 b	0.47	27.52 a	12.50 a	8.50 ab	16.17
300µm	0.40 a	0.52 a	0.48 a	0.46	9.61 b	10.09 ab	9.94 a	9.88
450µm	0.68 a	0.31 a	0.25 b	0.41	9.58 b	2.80 b	3.21 b	5.20
MEBS	2.70^**^	1.47^**^	1.29^**^	1.82	10.15	2.08	3.70	5.31

RPA: Means followed by a different lowercase letter in the columns are significantly different from each other by the Tukey’s test (p < 0.05). MEBS: ** = means significantly different from treatments using RPA by the Dunnett’s test (p < 0.01).

The application ranges of 7.0 and 9.0 m presented higher mean droplet density for all ESCLs, with a significant mean increase of approximately 2.0-fold in the quantity of droplets cm^-2^ compared to the 11.0 m application range (**Table 3**). However, the 9.0 m application range had a more uniform distribution in all canopy layers, but not differing statistically from the 7.0 m application range. Droplet size and droplet density were inversely correlated in all layers. Droplet sizes of 150 and 300µm presented higher means compared to 450µm, with increases of 6.29 droplets cm^-2^, equivalent to 67.84% and 47.36% respectively (**Table 3**).

Using the manual electric backpack sprayer (MEBS) resulted in a significantly higher mean droplet coverage, which was 65.0% higher than that found using the RPA (**Table 3**). Although the RPA treatments showed a lower mean droplet density, statistical analyses did not show a significant difference compared to the control treatment.

**Figure 6:**
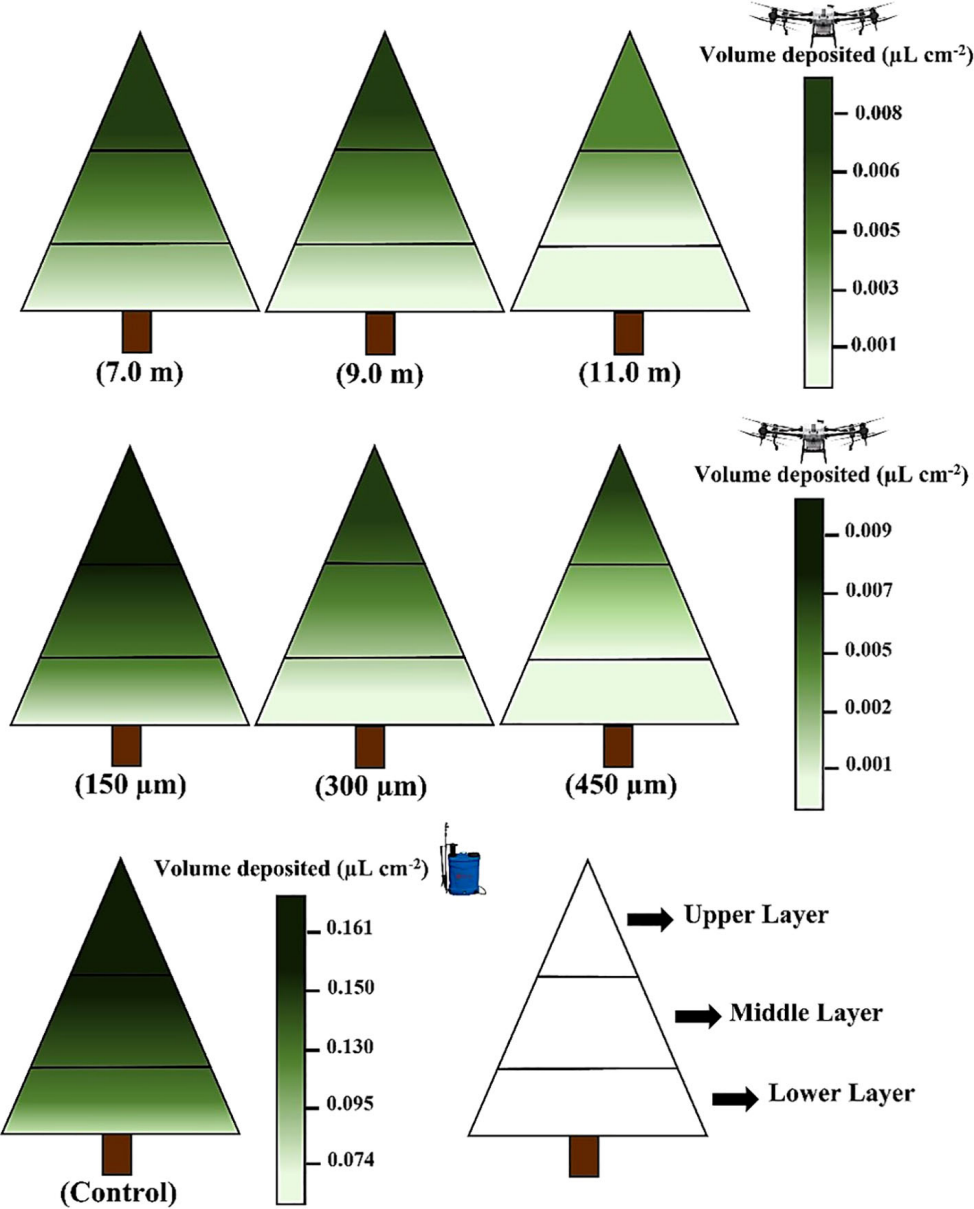
Droplet deposition (µL cm^-2^) on the upper, middle, and lower layers of the eucalyptus sprout canopy using a remotely piloted aircraft and a manual electric backpack sprayer (control); color gradient: dark green (higher deposition) to light green (lower deposition). Effects of factors in RPA application treatments analyzed separately: application range (7, 9, and 11 m) and droplet size (150, 300, and 450µm).

The application ranges of 7.0 and 9.0 m presented a higher droplet deposition on all ESCLs, whereas the 11.0 m application range resulted in the lowest droplet deposition, accumulating primarily on the upper ESCL (0.007µL cm^-2^) (**Figure 6**). The smallest droplet size (150µm) resulted in the highest droplet deposition on the upper (0.009µL cm^-2^), middle (0.006µL cm^-2^), and lower (0.003µL cm^-2^) ESCL compared to those found for 300 and 450µm. However, no significant difference within application ranges and droplet sizes was found for RPA, as shown by Anova (p ≥ 0.05).

**Figure 7:**
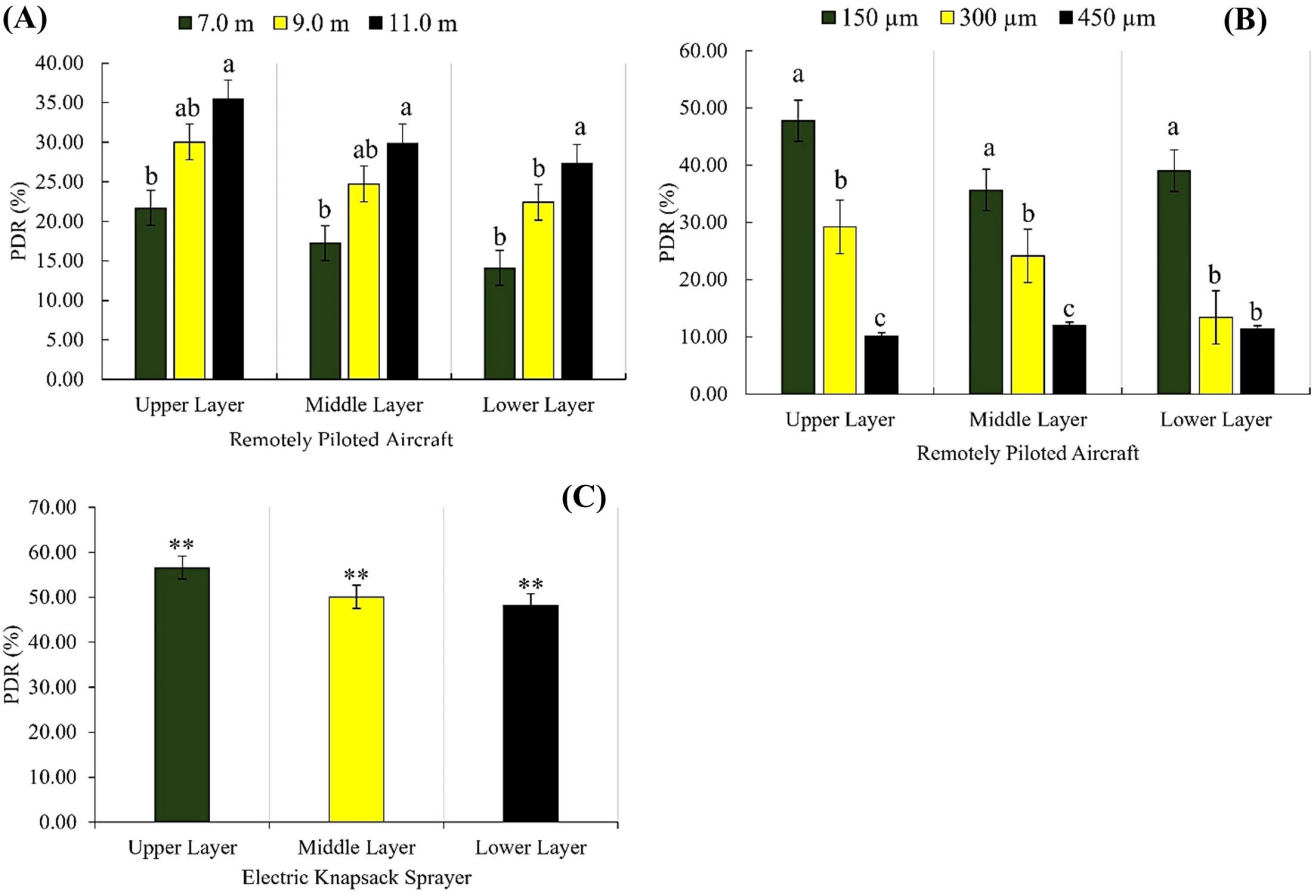
Potential drift risk (PDR) (%) in the upper, middle, and lower layers of the eucalyptus sprout canopy using a remotely piloted aircraft (**A**, **B**) and a manual electric backpack sprayer **(C)**. Factor effects analyzed separately: **(A)** application range (m) and **(B)** droplet size (µm). RPA: bars with different letters within canopy layers are significantly different from each other by the Tukey’s test (p < 0.05). MEBS: ** Means significantly different from RPA treatments by Dunnett’s test (p < 0.01).

Droplet deposition showed a consistent trend with droplet coverage and droplet density, regardless of the factors analyzed for the RPA treatments (application range and droplet size). The upper and middle ESCL presented higher droplet deposition compared to the lower layer (**Figure 7**). MEBS resulted in higher droplet deposition on the upper (0.161µL cm^-2^), middle (0.095µL cm^-2^), and lower (0.074µL cm^-2^) ESCL (**Figure 7**). For example, the highest deposition of 150µm droplets found using RPA for the upper (0.009µL cm^-2^), middle (0.006µL cm^-2^), and lower (0.003µL cm^-2^) ESCL were significantly different from those found using the MEBS (control), which was 94.40%, 93.68%, and 95.94% higher, respectively.

The 11.0 m application range resulted in the highest potential drift risk (PRD) for all ESCLs when using RPA, with significant differences of 38.82% and 15.33% higher compared to application ranges of 7.0 and 9.0 m, respectively. Contrastingly with the application ranges, which showed a directly proportional relationship with PRD, droplet size presented an inversely proportional relationship. The smallest droplet (150µm) resulted in a higher PRD for all ESCLs, whereas the largest droplet (450µm) showed a PRD lower than 13% for all layers (**Figure 7**).

The control treatment (MEBS) showed the highest mean PRD for all ESCLs compared to treatments using RPA. The overall mean PRD for MEBS in all ESCLs was 51.65%, denoting a significant PDR during the applications, significantly different from treatments using RPA.

### 3.2 Volumetric distribution by drop size class (DV_0.5_, DV_0.1_, DV_0.9_)

The application range significantly affected relative amplitude, but only in the middle and lower ESCL. The Anova indicated significant differences (p ≤ 0.001 and p ≤ 0.05) only for droplet size, volume distribution by droplet size class, and relative amplitude among ESCLs (**Table 4**).

**Table 4:** Analysis of variance for volume distribution by droplet size class (DV_0.5_, DV_0.1_, DV_0.9_) and relative amplitude (RA) in the upper, middle, and lower layers of eucalyptus sprout canopies when using a remotely piloted aircraft.

Factor	DV_0.5_			DV_0.1_		
	Upper	Middle	Lower	Upper	Middle	Lower
Range (R)	0.85 ^ns^	0.87 ^ns^	0.16 ^ns^	0.24 ^ns^	0.90 ^ns^	0.35 ^ns^
Droplet (D)	<0.001^***^	<0.01^**^	<0.001^***^	<0.001^***^	<0.01^**^	<0.001^***^
R × D	0.52 ^ns^	0.21 ^ns^	0.08^ns^	0.98^ns^	0.38^ns^	0.06^ns^
CV (%)	24.47	27.15	17.34	23.93	35.26	30.62
Factor	DV_0.9_			RA		
	Upper	Middle	Lower	Upper	Middle	Lower
Range (R)	0.44^ns^	0.27^ns^	0.35 ^ns^	0.17^ns^	<0.05^*^	<0.01^**^
Droplet (D)	<0.01^**^	<0.01^**^	<0.01^**^	<0.01^**^	<0.05^*^	<0.001^***^
R × D	0.98^ns^	0.14^ns^	0.50^ns^	0.74^ns^	0.48^ns^	0.23^ns^
CV (%)	26.72	23.68	16.53	35.53	33.21	28.94

Range = application range (m); Droplet = droplet size (µm); R × D = interaction between application range and droplet size. * = significant at p < 0.05; ** = significant at p < 0.01; *** = significant at p < 0.001; ns = not significant; CV (%) = coefficient of variation.

The application range showed no significant effect on variables related to volume distribution by droplet size class. DV_0.5_, DV_0.1_, and DV_0.9_ were significantly and directly proportional to droplet size. Thus, individually, the larger the droplet size set on the RPA control (represented by DV_0.5_) during the experimental treatments, the greater the mean droplet sizes (150, 300, and 450µm). The 9.0 m application range and 150µm droplet size combination presented the highest mean relative amplitude for all ESCLs, which directly affected the uniformity of droplet distribution.

Using the MEBS showed significant differences in variables related to volume distribution by droplet size class compared to treatments using RPA, exceeding 50% increase in these variables. However, the differences in mean relative amplitude were not significantly different in the different ESCLs when using RPA.

**Figure 8:**
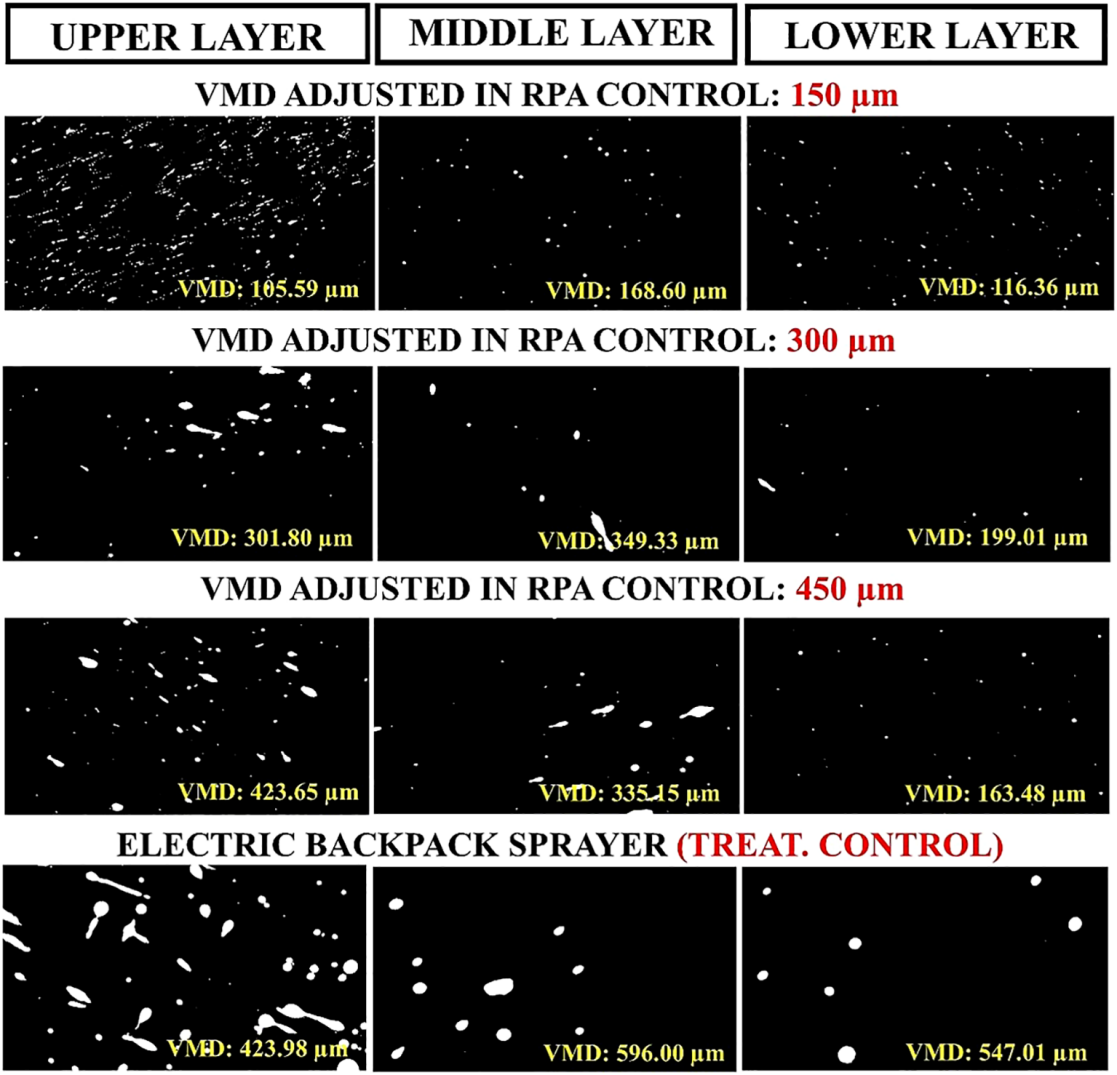
Droplet distribution in water-sensitive paper cards (representative of each treatment) using a remotely piloted aircraft (RPA) (regardless of application ranges) and a manual electric backpack sprayer (control treatment) in different eucalyptus sprout canopy layers (considering inner and outer canopy sections). VMD = volume median diameter or DV_0.5_.

Considering the droplet size of DV_0.5_, or volume median diameter (VMD), the means of canopy layers for the smallest droplet size set on the RPA control (150µm) was the closest to the actual droplet size (179.02µm) (**Table 5**). However, the mean DV_0.5_ of 300 and 450µm droplets differed from those set on the RPA during the experimental treatments. These results were confirmed by the analysis of droplet distribution using water-sensitive papers, considering the different treatments applied to the different eucalyptus canopy layers using RPA and MEBS (**Figure 8**).

**Table 5:** Mean DV_0.5_, DV_0.1_, DV_0.9_ (µm) and relative amplitude (RA) in eucalyptus sprout canopy layers (ESCL) (upper, middle, and lower) using a remotely piloted aircraft (RPA) and a manual electric backpack sprayer (MEBS).

	DV_0.5_				DV_0.1_			
RPA application range (m)								
ESCL	Upper	Middle	Lower	Mean	Upper	Middle	Lower	Mean
7.0 m	204.19a	211.78a	241.26a	219.07	116.64a	137.54a	145.15a	133.11
9.0 m	211.87a	223.45a	217.11a	217.47	128.09a	126.91a	135.25a	130.31
11.0 m	216.3a	222.22a	249.34a	230.00	138.60a	138.33a	170.00a	148.97
RPA droplet size (µm)								
150µm	154.90b	174.66b	179.02b	170.00	92.05b	103.21b	101.68 c	98.98
300µm	206.15b	225.05ab	238.26b	223.15	120.0b	131.50ab	145.80b	132.43
450µm	271.32a	257.75a	290.43a	273.16	171.40a	167.96a	203.93a	181.09
MEBS	448.93^**^	567.00^**^	437.81^**^	484.58	260.0^**^	328.75^**^	295.90^**^	294.88
	DV_0.9_				RA			
RPA application range (m)								
	Upper	Middle	Lower	Mean	Upper	Middle	Lower	Mean
7.0 m	285.03a	263.83a	294.58a	281.14	0.87a	0.65ab	0.64ab	0.72
9.0 m	328.14a	306.26a	296.78a	310.39	0.98a	0.83a	0.82a	0.87
11.0 m	301.95a	271.00a	316.72a	296.55	0.73a	0.58b	0.58b	0.63
RPA droplet size (µm)								
150µm	245.15b	248.93b	261.78b	251.95	0.96a	0.78a	0.88a	0.87
300µm	290.83b	290.60a	308.19ab	296.54	0.83ab	0.75ab	0.70a	0.76
450µm	380.0a	301.60a	338.10a	340.00	0.78b	0.52b	0.46b	0.59
MEBS	553.42^**^	664.96^**^	521.97^**^	580.11	0.68	0.54	0.51	0.58

RPA: Means followed by a different lowercase letter in the columns are significantly different from each other by the Tukey’s test (p < 0.05). MEBS: ** = means significantly different from treatments using RPA by the Dunnett’s test (p < 0.01).

### 3.3 Occupational exposure of applicators

**Figure 9:**
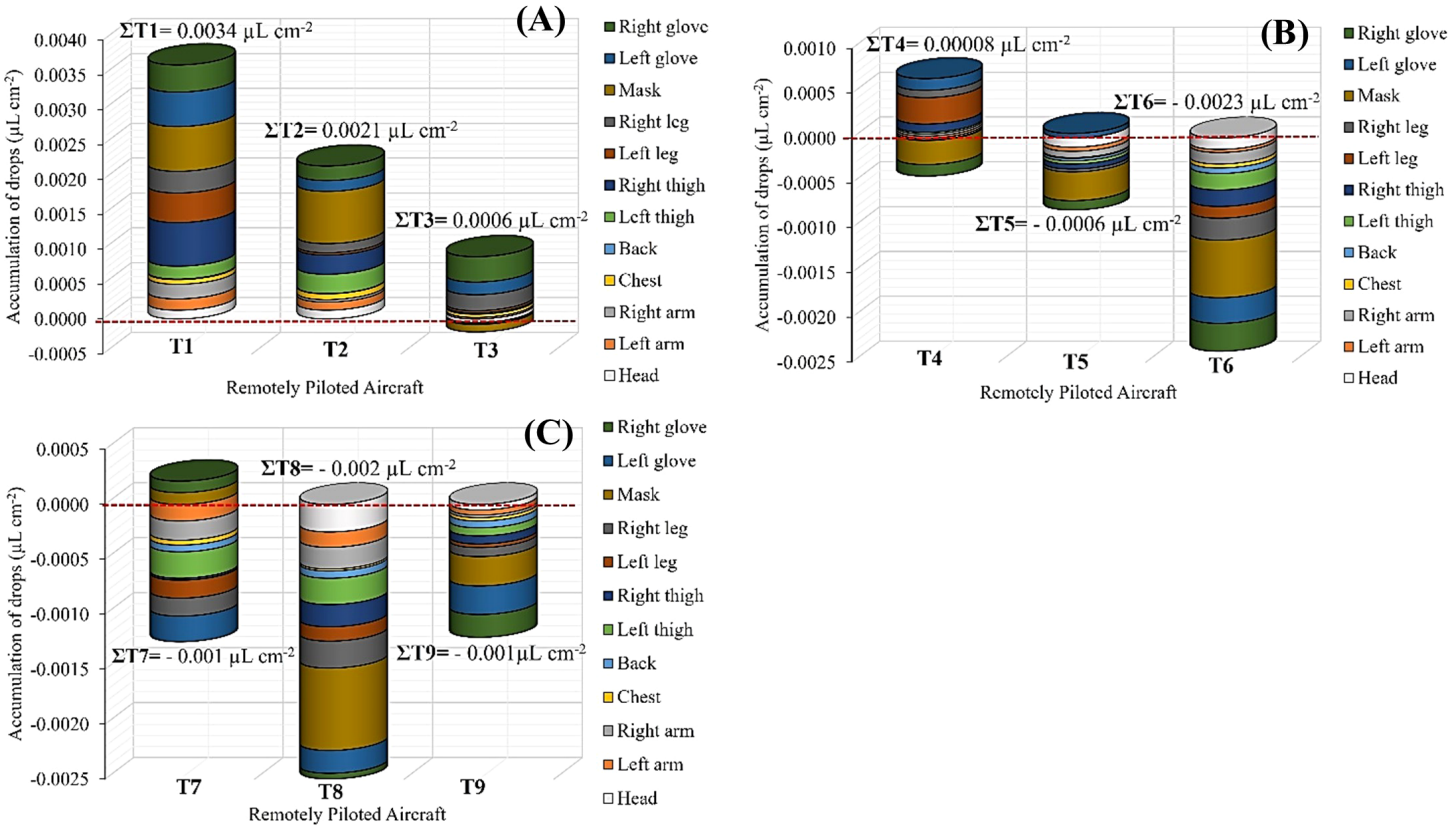
Occupational exposure of applicators (different body parts) using a remotely piloted aircraft and sum for the treatments evaluated (∑T) expressed as accumulation of droplets (µL cm-2). Treatments (droplet size × application range): **(A)** - T1 = 150µm × 7.0 m; T2 = 150µm × 9.0 m; T3 = 150µm × 11.0 m; **(B)**- T4 = 300µm × 7.0 m; T5 = 300µm × 9.0 m; T6 = 300µm × 11.0 m; **(C)**- T7 = 450µm × 7.0 m; T8 = 450µm × 9.0 m; and T9 = 450µm × 11.0 m.

Regarding the risk of occupational exposure for applicators when using RPA, treatments with 150µm droplets (T1, T2, and T3) showed higher accumulation of droplets on upper limbs, regardless of application ranges (**Figure 9**). This is confirmed by the highest total mean accumulated volume in the different body parts of applicators in T1, T2, and T3. For example, the 150µm droplet size with application ranges of 7.0 m (T1) and 9.0 m (T2) resulted in a higher volume deposited on the gloves, respirator, and legs, respectively. In the 11.0 m application range (T3), the deposits were limited to legs and gloves. This result was also found for treatment T4, which consisted of using 300µm droplets and 7.0 m application range.

Droplet depositions on different body parts are below the spectrophotometer’s detection limit, as evidenced in T4, indicating that the null absorbance readings were due to the low accumulation of droplets on different parts of applicators’ bodies when using RPA. This was found in all treatments using the largest droplets on the RPA (T7, T8, and T9).

**Figure 10:**
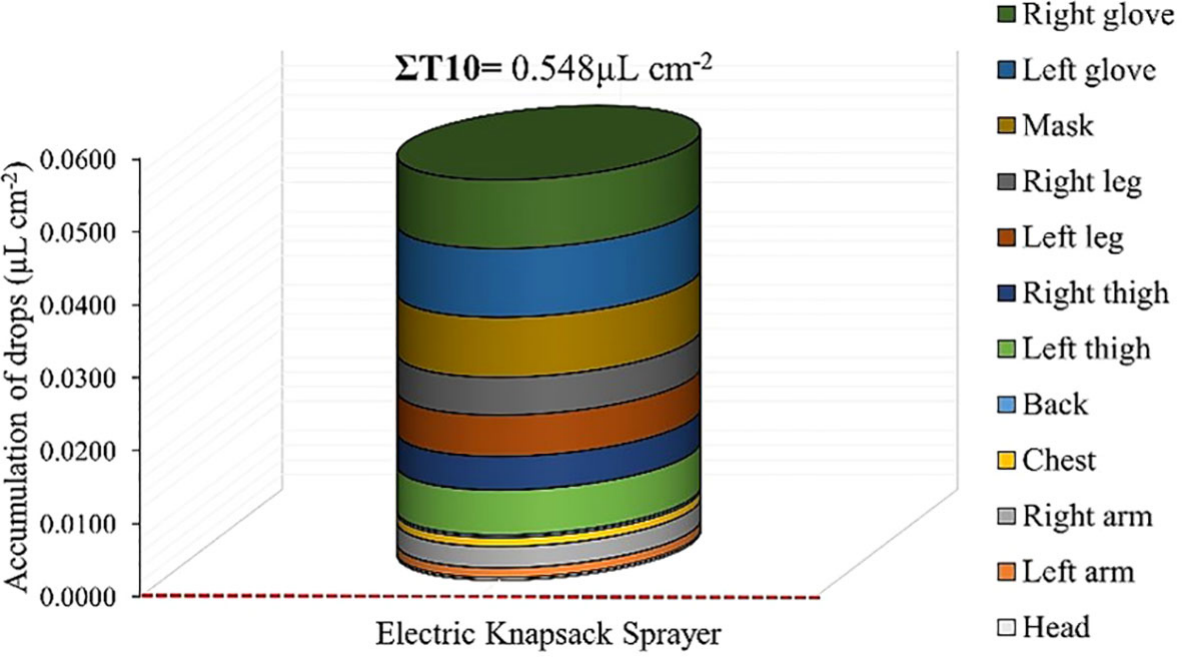
Occupational exposure of applicators (different body parts) using a remotely piloted aircraft and sum for the treatments evaluated (∑T) expressed as accumulation of droplets (µL cm^-2^) for a manual electric backpack sprayer (control, T10).

Using an MEBS (control) resulted in a higher accumulation of droplets on all body parts compared to treatments using RPA (**Figure 10**). The highest deposited volumes were found in gloves, respirator, legs, thighs, and back. Comparing the total highest volume accumulated in all body parts found for the control treatment and the RPA treatment 1 (0.0034µL cm^-2^), MEBS presented deposits 0.544µL cm^-2^ (99.37%) higher, corresponding to approximately 160-fold higher deposits of droplets on the applicators’ bodies, thus increasing the risk of occupational exposure for applicators.

## 4 Discussion

This study showed the effect of theoretical application range used in the RPA during the evaluations, differently from other studies that traditionally approach application ranges to evaluate the droplet spectrum on total and effective ranges in agricultural crops (Xiao et al., 2020; Cao et al., 2021; Dengeru et al., 2022) and weeds (Ahmad et al., 2020). Eucalyptus plants have higher foliage in the middle and lower layers, explaining the higher means for droplet coverage, density, and deposition on the upper and middle layers of eucalyptus sprout canopies and lower means for the lower layer. Previous studies on RPAs have demonstrated their effects on olive (Martinez-Guanter et al., 2020), common hazel (Özyurt et al., 2022), and apple (Wang et al., 2023) trees. Moreover, there was no sufficient statistical evidence of significant difference between inner and outer sections of the eucalyptus canopy for any treatment due to the little difference in leaf density between canopy sections, contrasting with results found for guava (Verma et al., 2022) and citrus (Yan et al., 2023) trees.

The highest mean droplet coverage, density, and deposition found for the 7.0 and 9.0 m application ranges indicated a higher application quality and uniformity in droplet density, as well as a lower potential drift risk (PRD) for the different ESCLs compared to the highest application range (11.0 m). However, the choice of the RPA application range affects the operational efficiency, flight speed, and droplet overlapping, explaining the highest mean droplet density and deposition. Therefore, reducing the application range results in greater droplet deposition; however, operational efficiency is decreased due to the need for frequent stops for tank refilling and battery changes when using RPAs.

Droplets of 150µm and 300µm maximized droplet coverage and penetration, but significantly increased PRD throughout the eucalyptus canopy compared to 450µm droplets, which decreased PRD to ≤ 13%, despite limiting droplet deposition on the upper canopy layer. Shan et al. (2021) and Wang et al. (2024) found similar results. Smaller droplets generally result in better droplet coverage and density, increasing droplet penetration into shrubby crops, which is desirable for applications of contact-action pesticides. However, these droplets are susceptible to drift and evaporation under unfavorable weather conditions (Chen S. et al., 2020; Ribeiro et al., 2023b). Conversely, larger droplets have higher kinetic energy due to their greater mass and speed, minimizing the impacts of primary drift, but maximizing canopy effects, limiting coverage in the lower layers (Li et al., 2021; Vitória et al., 2022; Shan et al., 2022; Ribeiro and Vitória, 2024).

The eucalyptus stumps in the experimental area had numerous sprouts, resulting in a rounded canopy architecture with high leaf density, significant branch and leaf overlaps, reaching a mean canopy height of 2.80 m. Therefore, the experimental challenge was high, as chemical control using herbicides is the primary method for eradication of eucalyptus sprouts in eucalyptus forestry. However, sufficient sprout height and leaf area for absorption and translocation of herbicides should be considered for a higher control efficiency (Ferreira et al., 2010). In addition, eucalyptus sprouting capacity is influenced by its genetic potential and the presence of lignotubers (Ferrari et al., 2005). These factors are essential for selecting the appropriate operational parameters for RPA operation during herbicide application planning for eucalyptus sprouts.

Volume distribution by droplet size class was strongly affected by droplet size. However, the RPA used had centrifugal spray nozzles with multiple radial grooves in the internal disc wall. The centrifugal distributor operates based on the centrifugal force generated by the rotation of the rotor during flight. This rotation propels the liquid, breaking it into droplets that are directed over the area to be sprayed.The main characteristic of this type of spray nozzle is the possibility of adjusting the droplet size by varying rotation speed; high rotation speeds generate finer droplets, whereas lower speeds produce larger droplets (Chen et al., 2022; Nascimento and Vitória, 2022; Crause et al., 2023). One of the main advantages of this nozzle type is the reduced clogging risk, making it adequate for spraying low-solubility pesticide and foliar fertilizer solutions (Qingqing et al., 2017; Wang et al., 2020). However, the droplet spectrum classification generated by these nozzles in RPAs is not yet fully understood and, currently, consolidated norms for spray nozzles are applied for their classification (ASABE, 2020; ISO, 2018).

According to the DV_0.5_, or volume median diameter, obtained for the different layers of the eucalyptus sprout canopy, only 150µm and 300µm droplets were similar to the actual droplet size set on the RPA control, as only the droplets with these sizes easily penetrated into the eucalyptus sprout canopy. Additionally, the downwash effect, which is the air flow generated by the RPA multi-rotors, contributed to increases in low-diameter droplets (Wang et al., 2023) by the turbulence under the eucalyptus sprout canopy. Typically, the results of droplet size distribution are poorly evidenced in studies on RPAs with centrifugal nozzles; thus, the droplet size set on an RPA with centrifugal nozzles does not always correspond to the droplet size that reaches the target, as the distance between the spray nozzle and the target becomes greater, directly affecting droplet lifespan (Wang et al., 2018).

The control treatment, using a manual electric backpack sprayer (MEBS), showed a higher droplet coverage and deposition on all canopy layers. However, droplet density was lower than those obtained in treatments using the RPA. These results can be attributed to the combination of a high application rate (100 L ha^-1^) and the use of flat-fan spray nozzles, which results in the formation of extremely coarse droplets, reduces droplet density, and significantly increases internal drift risk, according to results reported by Ribeiro et al. (2023b). However, the high droplet coverage, density, and deposition provided by ground sprayers do not always lead to greater target control, as RPAs have smaller tank capacities and reduced application rates, resulting in extremely concentrated droplets (Wang et al., 2019; Vitória et al., 2023). Furthermore, the ergonomical restriction posed to applicators during spraying is one of the main challenges of backpack sprayers, mainly on mountainous terrain.

The use of RPAs for agricultural applications is one of the few methods in which the applicator operates remotely, outside the spraying zone, ensuring the operational safety and efficiency. The results of the present study showed that the accumulation of droplets on applicators using a RPA is concentrated on upper limbs, hands, respirator device, and legs, primarily by 150µm droplets, regardless of application ranges. Recently, Lan et al. (2024) evaluated the use of RPAs in mango trees and found that fine droplets resulted in less contamination of applicators compared to medium and coarse droplets, which accumulated mainly on lower limbs and different RPA parts. Wang et al. (2024) evaluated applications on coconut palms and found that most droplets deposited on applicators were on the upper part of their bodies, mainly on the chest. Additionally, Dubuis et al. (2023) evaluated applications on an apple orchard and found the highest accumulation of droplets on legs, followed by chest, back, and thighs. Thus, the results of previous studies differ from those found in the present study, denoting variability in the accumulation of droplets on applicators depending on the canopy architecture, distance of applicators from the target area, weather conditions, and operational parameters defined for the RPA.

Regarding the risk of exposure for applicators when using MEBS, the high mean accumulation of droplets on applicators’ body parts was due to the longer exposure time to sprayed solution. The applicator held the sprayer lance and move it from top to bottom of the canopy on both sides the planting row due to the height of the eucalyptus sprout canopy, as typically done during the control of eucalyptus sprouts. Therefore, higher accumulation of droplets was found on the applicators’ upper limbs due to increased hand exposure, reaching approximately a 160-fold higher accumulation compared to the RPA. Yan et al. (2021) and Kuster et al. (2023) found similar results, with significant decreases (>90%), when comparing RPA and MEBS. However, the exposure to pesticides can occur through several routes in the body, including dermal (contact with the skin) respiratory (inhalation of vapors, particles, or droplets), oral (accidental ingestion), and ocular (splashing into mucous membranes). Therefore, the use of personal protective equipment before, during, and after applications of pesticides is essential, regardless of the application technique used (ground or aerial) (Tsakirakis et al., 2014; Cao et al., 2015).

Although the results of the present study demonstrate for the first time the quality of droplet distribution in eucalyptus sprouts and the lower risk of exposure for applicators using RPA compared to MEBS, further studies are needed to confirm the efficiency for applications of chemical or biological pesticides and foliar fertilizers. Moreover, additional operational parameters that can be set on RPAs (operational flight height and speed, application rates, and droplet size) should be tested in different phenological stages of eucalyptus plants, comparing to conventional application methods, focused on enhancing results related to application efficiency. Thus, efficient and safe use of RPAs in forestry will be possible, as already reported by Leroy et al. (2019) and Pachuta et al. (2023), aligned with the United Nations (UN) Sustainable Development Goals (SDGs), specifically the SDG 2 (Hunger Zero and Agriculture) and SDG 15 (Life on Land), established to promote the sustainable protection of crops and planted forests, while contributing to global environmental security (Pranaswi et al., 2024).

## 5 Conclusions

The interaction between factors (application range and droplet size) was not significant for application quality, droplet deposition, and occupational exposure risk for applicators when using a remotely piloted aircraft (RPA).

Setting the RPA to application ranges of 7.0 and 9.0 m with droplet sizes of 150µm and 300µm resulted in higher quality of droplet distribution throughout eucalyptus sprout canopies. However, it increased the risk of occupational exposure for applicators, although the risk was not significant compared to the use of a manual electric backpack sprayer.

Setting the RPA to droplet size of 450µm resulted in droplets concentrated mainly on the upper and middle layers of eucalyptus sprout canopies, and decreased the potential drift risk (≤ 13%).

The use of a manual electric backpack sprayer resulted in lower uniformity in droplet distribution throughout eucalyptus sprout canopies and increased the risk of occupational exposure for applicators by up to 99.37% compared to the use of an RPA.

## Data Availability

The datasets presented in this article are not readily available because Private company research and experiment. Requests to access the datasets should be directed to luis.f.ribeiro@edu.ufes.br.
